# Involvement of Wnt7a in the role of M2c microglia in neural stem cell oligodendrogenesis

**DOI:** 10.1186/s12974-020-01734-3

**Published:** 2020-03-19

**Authors:** Miriam Mecha, Natalia Yanguas-Casás, Ana Feliú, Leyre Mestre, Francisco Javier Carrillo-Salinas, Kristoffer Riecken, Diego Gomez-Nicola, Carmen Guaza

**Affiliations:** 1 Departamento de Neurobiología Funcional y de Sistemas, Grupo de Neuroinmunología, Instituto Cajal, CSIC, Madrid, Spain; 2 Present address: Grupo de Investigación en Linfomas, Instituto Investigación Sanitaria Puerta de Hierro-Segovia de Arana (IDIPHISA), Majadahonda, Madrid, Spain; 3 Tufts University School of Medicine, Boston, USA; 4 Research Department Cell and Gene Therapy, Clinic for Stem Cell Transplantation, University Medical Centre Hamburg-Eppendorf, Hamburg, Germany; 5 Centre for Biological Sciences, University of Southampton, Southampton, UK

**Keywords:** Microglia, Neural stem cells, Wnt, β-Catenin, Oligodendrogenesis, Subventricular zone

## Abstract

**Background:**

The participation of microglia in CNS development and homeostasis indicate that these cells are pivotal for the regeneration that occurs after demyelination. The clearance of myelin debris and the inflammatory-dependent activation of local oligodendrocyte progenitor cells in a demyelinated lesion is dependent on the activation of M2c microglia, which display both phagocytic and healing functions. Emerging interest has been raised about the role of Wnt/β-catenin signaling in oligodendrogenesis and myelination. Besides, cytokines and growth factors released by microglia can control the survival, proliferation, migration, and differentiation of neural stem cells (NSCs), contributing to remyelination through the oligodendrocyte specification of this adult neurogenic niche.

**Methods:**

TMEV-IDD model was used to study the contribution of dorsal SVZ stem cells to newly born oligodendrocytes in the corpus callosum following demyelination by (i) *en*-*face* dorsal SVZ preparations; (ii) immunohistochemistry; and (iii) cellular tracking. By RT-PCR, we analyzed the expression of Wnt proteins in demyelinated and remyelinating corpus callosum. Using in vitro approaches with microglia cultures and embryonic NSCs, we studied the role of purified myelin, Wnt proteins, and polarized microglia-conditioned medium to NSC proliferation and differentiation. One-way ANOVA followed by Bonferroni’s post-hoc test, or a Student’s *t* test were used to establish statistical significance.

**Results:**

The demyelination caused by TMEV infection is paralleled by an increase in B1 cells and pinwheels in the dorsal SVZ, resulting in the mobilization of SVZ proliferative progenitors and their differentiation into mature oligodendrocytes. Demyelination decreased the gene expression of Wnt5a and Wnt7a, which was restored during remyelination. In vitro approaches show that Wnt3a enhances NSC proliferation, while Wnt7a and myelin debris promotes oligodendrogenesis from NSCs. As phagocytic M2c microglia secrete Wnt 7a, their conditioned media was found to induce Wnt/β-Catenin signaling in NSCs promoting an oligodendroglial fate.

**Conclusions:**

We define here the contribution of microglia to Wnt production depending on their activation state, with M1 microglia secreting the Wnt5a protein and M2c microglia secreting Wnt7a. Collectively, our data reveal the role of reparative microglia in NSC oligodendrogenesis with the involvement of Wnt7a.

## Background

In the adult brain, it is essential that new oligodendrocytes are continuously generated due to their role in preserving saltatory conduction, and in the metabolic and trophic support they offer to neurons. During rodent brain development, precursors in the ventral and dorsal telencephalon give rise to oligodendrocytes and to oligodendrocyte progenitor cells (OPCs [1];). Moreover, there is evidence that oligodendrocytes can be generated postnatally from subventricular zone (SVZ) progenitors [2]. The SVZ is a specialized niche in the walls of the lateral ventricles of the forebrain that contents multipotent cells known as neural stem cells (NSCs), cells that can self-renew and differentiate into neurons, astrocytes, or oligodendrocytes [3, 4]. Adult NSCs are slowly dividing progenitors referred to as B1 cells, and they generate transit amplifying progenitors (type-C cells) that in turns differentiate into neuroblasts (type-A cells). These neuroblasts generally migrate to the olfactory bulb via the rostral migratory stream (RMS) to replace local interneurons [5].

Beside major contribution of local OPCs to remyelination, it appears that the oligodendrogenic potential of the SVZ is enhanced in response to demyelination, generating new oligodendrocytes in the corpus callosum, fimbria fornix, and striatal white matter tracts [6–14]. Specifically, oligodendrogenic signals are enriched in the dorsal SVZ relative to the lateral SVZ, controlling the expression of the Olig2 and Sox10 transcription factors, and Wnt/β-Catenin signaling [15]. Given the proximity of this niche to the white matter tracts of the corpus callosum, the capacity of this zone to generate oligodendrocytes may be a result of its physical location.

There is increasing evidence that microglia fulfill an essential role in postnatal neurogenesis in the adult dentate gyrus, participating in active phagocytosis [16] and cytokine release upon activation [17], thereby influencing SVZ neurogenesis, and supporting the migration and survival of neuroblasts along the rostral migratory stream (RMS) [18]. Microglia enhance NSC neurogenesis in vitro [19, 20], and they promote the migration and differentiation of embryonic NSCs [21, 22]. Indeed, microglia are pivotal cells in remyelination, mediating the phagocytosis of myelin debris, OPC recruitment, and their differentiation [23, 24]. Hence, microglia could control adult oligodendrogenesis upon demyelination, targeting local OPC populations and NSC progenitors in the SVZ.

Here, we analyzed the generation and maturation of oligodendrocytes from NSCs and the possible implication of microglial cells in these processes, acting via Wnt signaling. To study the contribution of the dorsal SVZ to remyelination, we used the Theiler’s murine encephalomyelitis virus-induced demyelinating disease (TMEV-IDD) model of multiple sclerosis (MS), in which spontaneous remyelination in the corpus callosum is driven by OPC mobilization and differentiation, and M2c microglia [25]. The TMEV model is a viral model of progressive MS with a preclinical phase with prominent demyelination in the corpus callosum and other brain areas at 35 days post-infection (dpi) due to a pro-inflammatory reaction against the virus (acute encephalomyelitis). When the antiviral reaction diminishes, endogenous mechanisms of repair begin and partial remyelination is evident at 60 dpi which is subsequently lost with progression into the chronic phase of the disease with demyelination and axonal damage mainly in the spinal cord and with the onset of the motor disability [26]. We found that corpus callosum demyelination was paralleled by an increase in B1 cells and of pinwheels in the dorsal SVZ in vivo, contributing to the generation of new mature oligodendrocytes. The expression of genes in the canonical (Wnt3a, Wnt7a) and non-canonical (Wnt5a) Wnt/β-Catenin signaling pathway was dysregulated in the demyelinated corpus callosum and during remyelination. In the TMEV paradigm, we speculate that Wnt signals and the SVZ-derived cells are surrounded not only by inflammatory microglia, dead oligodendrocytes, myelin debris, axonal damage but also by astrocyte activation and peripheral immune cell recruitment among others. As myelin removal is a critical step in the remyelination process [27] and microglia are actively involved in the clearance of myelin debris [28], we also focus in the in vitro culture approaches dissecting the scenario to the four players that we propose: microglia, myelin debris, Wnt proteins, and NSC. Thus, we explored whether the changes to the local environment associated with demyelination affect the generation and maturation of oligodendrocytes from NSCs in vitro, including the alterations in myelin, Wnt signaling, and microglia. We found that purified myelin affects Wnt5a but not Wnt7a expression in microglia, and that myelin directly promotes oligodendrogenesis from NSCs. Direct addition of the Wnt3a protein induced NSCs proliferation, whereas Wnt7a potentiated NSC oligodendrogenesis. For the first time, we show that microglia secretes Wnt proteins, and that their pattern secretion depends on the activation state of these cells. Indeed, conditioned media containing the Wnt7a protein secreted by M2c microglia promoted NSC oligodendrogenesis.

## Methods

### Animals and Theiler’s virus infection

SJL/J female mice were obtained from Envigo and maintained under standard conditions. Four- to six-week-old mice were injected intracranially with 2 × 10^6^ plaque forming units (pfu) of the TMEV Daniel’s strain in a volume of 30 μl Dulbecco’s minimal essential medium (DMEM) supplemented with 10% fetal calf serum (FCS: [29]). Sham-operated mice received the vehicle alone (30 μl). All experiments were performed following the ARRIVE guidelines, and in accordance with EU (Directive 2010/63/EU) and National guidelines (Royal Decree 53/2013 BOE No. 34 and Comunidad de Madrid: ES 280790000184). The Ethics Committee on Animal Experimentation at the Instituto Cajal (CSIC) approved all the procedures described in this study (protocol number: 2013/03 CEEA-IC).

### Stereotaxic injection of retroviral particles

Mice were anesthetized with isofluorane 33 days after TMEV infection and 1 μl of γ-retroviral-SFFV-Venus viral particles (LentiGO vectors, prepared and characterized as described in [30]) was injected stereotaxically into the right hemisphere at the Bregma coordinates: anteroposterior − 0.6 mm, lateral 1 mm, dorsoventral 1.8 mm.

### Whole mount dissections

The dorsal, medial, and lateral walls of the right brain ventricle were prepared as described previously [31]. After saline perfusion on day 35 post-infection (p.i.), the brains were fixed overnight at 4 °C in 4% paraformaldehyde (PFA)/0.1% Triton X-100. After staining, the ventricle walls were further dissected from the underlying parenchyma to obtain 200–300-μm-thick tissue sections that were mounted on a slide with Mowiol and coverslipped. Three animals were studied for each experimental group.

### Microglial cell cultures

Primary microglia cell cultures from P0–P2 (post-natal day 0–2) Wistar rats were prepared as described previously [32, 33], with minor modifications (Mecha et al. in doi: 10.1038/protex.2011.218, Open Nature Exchange protocol only online). Purified microglia were plated at a density of 100,000 cells/cm^2^ for PCR and ELISA analysis, and to obtain microglial conditioned media, or at 50,000 cells/cm^2^ for immunohistochemistry. Cells were maintained for 3 days at 37 °C and in an atmosphere of 5% CO_2_ in a defined medium containing 10% horse serum (HS) and 10% fetal bovine serum (FBS). The cells were then incubated for 1 h in serum-free DMEM prior to a 6- or 24-h exposure to the treatments prepared in distilled water: LPS (50 ng/ml); a combination of IL-4 and IL-13 (both at 10 ng/m;); or TGF-β1 (20 ng/ml).

### Neural stem cell cultures

Rat NSCs (N7744-100) were purchased from Invitrogen, isolated from the cortices of fetal E14 Sprague-Dawley rats; these NSCs retain the capacity for self-renewal and to differentiate into neurons, astrocytes, and oligodendrocytes [34]. Cells were expanded in Knockout DMEM:F12 medium (12660-012, Invitrogen) supplemented with 2 mM Glutamax (35050-061, Invitrogen), 2% Stempro® NSC SFM (A1050901, Invitrogen), 20 ng/ml bFGF (450-33, PeproTech), 20 ng/ml EGF (E5160, Merck) and antibiotics, and they were then plated at 50,000 cells/cm^2^ on poly-d-lysine coated coverslips (5 mg/ml, Merck) for immunocytochemical studies.

### Reagents

LPS from *Escherichia. coli* serotype 026:B6 (L3755) was purchased from Merck, human TGFβ1 (100-21), rat IL-4 (400-04), and rat IL-13 (400-16) were obtained from PeproTech, and mouse Wnt3a (1324-WN), human/mouse Wnt5a (645-WN), and mouse Wnt7a (3008-WN) were purchased from R&D Systems.

### Immunohistochemistry

Immunohistochemistry was performed on fixed tissue from six animals from each experimental group. The mice were anesthetized with pentobarbital (50 mg/kg body weight, i.p.) and perfused with saline on day 35 and 60 post-TMEV infection (p.i), and the brain of the mice was fixed overnight in 4% PFA prepared in 0.1 M phosphate buffer saline (PBS). Free-floating coronal vibratome sections of the brain (30 μm thick) were washed three times in PBS, incubated with PBS containing 0.1% Triton-X100 (PBT), and blocked for 1 h at room temperature in blocking buffer: PBT containing 5% normal serum (Vector Laboratories). Sections were then incubated overnight at 4 °C with primary antibodies against the following proteins: β-Catenin (1:400, 04-958, Merck), CC1 (1:200, OP80, Calbiochem), Doublecortin (DCX–1:500, SC-8066, Santa Cruz Biotechnologies), GFAP (1:1000, G9269, Merck), Iba-1 (1:1000, 019-19741, Wako), NG2 (1:500, AB5320, Merck), Olig2 (1:200, sc-19967, Santa Cruz Biotechnologies), and γ-Tubulin (1:200, sc-7396, Santa Cruz Biotechnologies). On the following day, the sections were rinsed three times with PBT and they were then incubated for 1 h with an Alexa Fluor-conjugated (1:500, Molecular Probes Inc.) secondary antibody diluted in blocking buffer. In all cases, the specificity of staining was confirmed by omitting the primary antibody.

### Immunocytochemistry

Cells were fixed in 4% PFA for 20 min and permeabilized in PBT. After blocking with 5% normal serum, cells were incubated for 2 h at room temperature with antibodies against the following proteins, all diluted in blocking buffer: β-Catenin (1:200, 04-958, Merck), β-III Tubulin (1:1000, ab7751, Abcam), BrdU (1:500, ab6326, Abcam), GFAP (1:1000, G9269, Merck), O4 (1:1000, MAB345, Merck), and OX-42 (1:1000, MCA275G, Serotec). After rinsing, the coverslips were incubated for 1 h at room temperature with Alexa Fluor-conjugated secondary antibodies (1:500, Molecular Probes, Inc.), which were then mounted on slides with Mowiol and kept in the dark at 4 °C. For BrdU analysis, NSCs were incubated with BrdU (10 μM) for 24 h in complete NSC medium with growth factors and in the presence of the different drugs. The cells were then fixed in 4% PFA for 20 min, treated with 2 N HCl for 10 min, blocked, and incubated with the antibody against BrdU (Abcam, 1:1000). After washing, the cells were incubated with Alexa Fluor-conjugated antibody (1:500, Molecular Probes, Inc.) and counterstained with DAPI.

### ELISA

The Wnt5a and Wnt7a in the supernatants of microglia cultures were measured using specific solid-phase sandwich ELISA kits (CSB-EL026138RA and CSB-EL026141MO, Cusabio) following the manufacturer’s recommendations (four independent experiments with three replicates were studied). The sensitivity of the assay for Wnt5a detection was 15.6 pg/ml, and the intra and inter coefficient of variation were 8% and 10%. The sensitivity of the assay for Wn7a detection was 0.156 ng/ml, and the intra and inter coefficient of variation were 8% and 10%.

### mRNA extraction, reverse transcriptase (RT)-PCR, and real-time PCR

For in vitro studies, microglial cells were seeded at a density of 100,000 cells/cm^2^ and they were lysed 6 h or 24 h after stimulation with known agents that induce the polarization of microglia to obtain M1 (LPS), M2a (IL-4 and IL-13), and M2c (TGFβ) microglia [35, 36]. In another subset of experiments, the microglia cells were lysed 6 h after the addition of purified myelin (2 μg/ml). Up to six independent experiments were performed on the microglia cultures. To analyze gene expression in vivo, the corpus callosum was isolated from the whole brain in ice-cold PBS on day 35 and 42 p.i., studying 6–10 animals per experimental group. The total RNA was extracted from the tissue using an RNeasy Lipid Tissue Mini Kit (QIAGEN), and it was treated with DNaseI (Promega) before 1 μg of this RNA was reverse transcribed using the high capacity cDNA reverse transcription kit (Applied Biosystems). The cDNA obtained was amplified by real-time PCR in a 7500 Real Time PCR System (Applied Biosystems) using the Taqman™ PCR Master Mix. Gene expression was determined with 7500 Software v2.0.4 applying the comparative Ct (cycle threshold) method. The ΔCt was calculated as the difference between the Ct of each target gene and the Ct of the RPS29/18S housekeeping gene. Taqman assays (ThermoFisher) were used to determine the mRNA expression of the following genes: mouse Wnt3a (Mm00437337_m1), mouse Wnt5a (Mm0437347_m1), mouse Wnt7a (Mm0437356_m1), mouse RPS29 (Mm02342448_m1), rat Wnt3a (Rn01470643_m1), rat Wnt5a (Rn01402000_m1), rat Wnt7a (Rn01425352_m1), and rat RPS29 (Rn00820645_m1).

### Myelin purification

Myelin from 9-week-old Wistar rat brains was isolated by sequential centrifugation on a discontinuous sucrose gradient, as described previously [25]. Briefly, 2 g of rat brain tissue was mechanically disaggregated in a 0.3 M sucrose solution, and then layered onto a sucrose gradient composed of 0.3 M and 0.83 M sucrose. Sequential ultracentrifugation was performed at 4 °C using a Thermo Scientific SureSpin 630 Rotor: 75,000 g, 30 min; 75,000 g, 15 min; and 12,000 g, 15 min. After two rounds of hypo-osmotic shock with a Tris-HCl buffer solution, the myelin was resuspended in sodium carbonate buffer (0.1 M NaHCO_3_–Na_2_CO_3_, pH 9.4: [37]) and stored at − 80 °C. The myelin protein content was determined by the Bradford method using bovine serum albumin as a standard.

### Image acquisition and analysis

All confocal images were acquired on a Leica TCS SP5 confocal microscope and for in vivo studies individual images of 4–5 sections were analyzed. Immunostaining and cell counts were quantified using Fiji software (designed by the National Institutes of Health), evaluating the proportion of staining relative to the total area in Sham animals and the total number of cells/mm^2^. In the case of DCX^+^ cells, three-dimensional images were created using the Imaris software using the “Absolute intensity” filter. In whole mount preparations, a single image of the surface of the dorsal, lateral, and medial wall of the cerebral ventricle was acquired, and up to three independent experiments from each experimental condition were assessed for in vitro analyses. Confocal stacks with 2 μm step size were captured in the z-direction from five fields per experimental condition and experiment, using a × 20 objective (three to four independent experiments were performed). The proportion of BrdU^+^ cells, neurons, astrocytes, and oligodendrocytes was evaluated using the cell counter Fiji plug-in. To assess oligodendrocyte maturation, the area occupied by O4 was analyzed and divided into three states of oligodendrocyte maturation: type 1, OPCs (0–500 μm^2^); type 2, immature oligodendrocytes (501–1500 μm^2^); and type 3, mature oligodendrocytes (above 1501 μm^2^). The same threshold was set for all the cells and all experimental conditions.

### Statistical analysis

GraphPad Prism 5.0 (GraphPad Software Inc., San Diego, USA) was used to analyze the data using either a one-way ANOVA followed by Bonferroni’s post-hoc test, or a Student’s *t* test, as appropriate: *p < 0.05; **p < 0.01; ***p < 0.001.

## Results

### Dorsal SVZ in the TMEV-IDD model

**Fig. 1:**
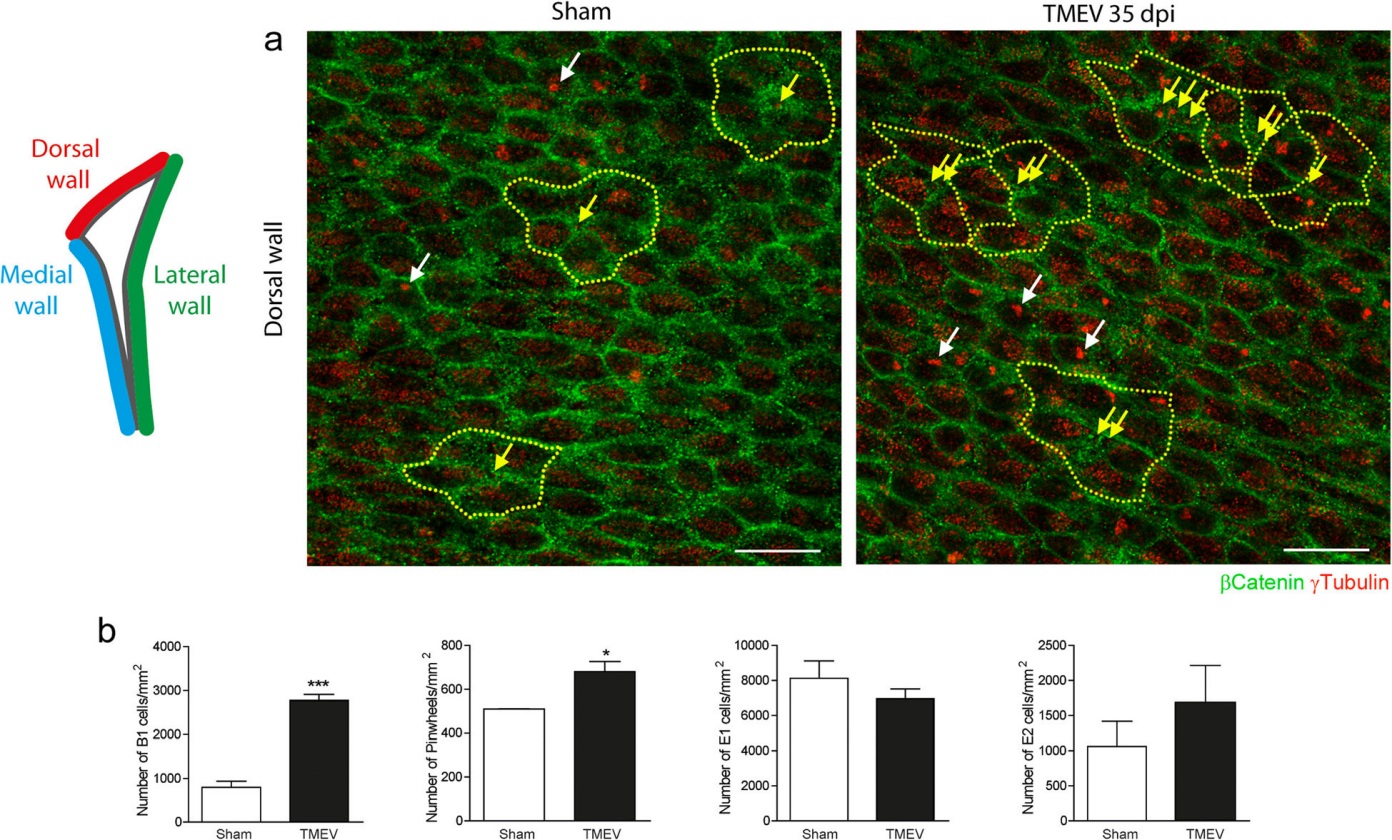
Alterations to cells in the dorsal SVZ in response to TMEV infection. **a** Confocal images of the *en*-*face* view of the dorsal lateral wall stained for γ-tubulin (red) and β-catenin (green). Pinwheels, identified by dashed lines, are comprised of B1 cells surrounded by ependymal E1/E2 cells. Yellow arrows indicate the apical surface of the B1 cells, and white arrows illustrate the ependymal E2 cells identified by two basal bodies. **b** Quantification data, *N* = 3 mice per group. Statistics: *p ≤ 0.05; ***p ≤ 0.001 TMEV 35 dpi (days post-infection) vs. Sham; Student’s *t* test. Scale bar: 20 μm

**Fig. 2:**
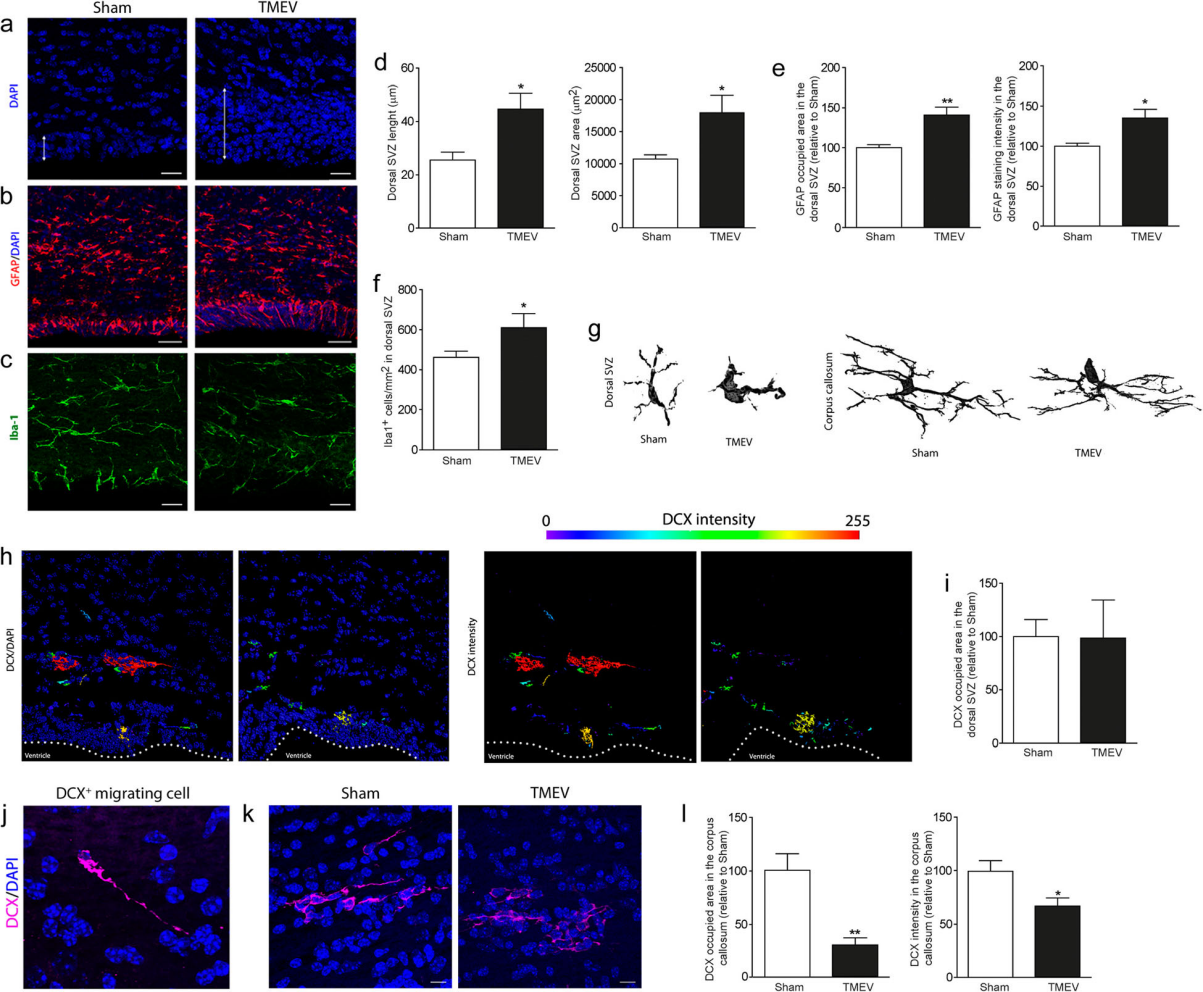
Changes in cell density in the dorsal SVZ and the adjacent corpus callosum at remyelination onset. **a**, **d** DAPI cell density, analyzed relative to the length (μm) and total area (μm^2^) of the dorsal SVZ. GFAP (**b**, **e**) and Iba1 (**c**, **f**) labelling in the dorsal SVZ supports the increase in the astrocyte (occupied area and intensity) and microglia (total number of microglia/mm^2^) cell density. **g** Morphological changes in microglia are evident in the dorsal SVZ but not in the adjacent demyelinated corpus callosum. **h** The intensity of DCX labelling in the dorsal SVZ and adjacent corpus callosum reconstructed by Imaris software. **i** DCX^+^ cell density in the dorsal SVZ showing no changes between the experimental groups. **j** Typical shape of a migrating DCX^+^ progenitor found in the corpus callosum and reconstructed by Imaris software. **k**, **l** Analysis of DCX^+^ cell density and the intensity of staining in the demyelinated corpus callosum. *N* = 6 mice per group. Statistics: *p ≤ 0.05; **p ≤ 0.01 vs. Sham; Student’s *t* test. Scale bar 10 μm in (**j**); 25 μm in (**a**, **c**); 50 μm in (**b**)

TMEV infection induces pronounced demyelination in the corpus callosum before clinical symptoms are evident (e.g., day 35 p.i.), which is followed by spontaneous yet incomplete remyelination (day 60 p.i.) that is no longer evident on progression into the chronic phase of the disease [38]. Corpus callosum demyelination is concomitant with an increase in GFAP+ type B astrocytes in the SVZ and increased proliferation of this area by day 35 p.i. [38]. *En*-*face* preparations of the dorsal ventricle wall (Fig. 1) confirmed an increase in the number of B1 cells and pinwheels in TMEV-infected mice (Fig. 1a, b), without affecting the number of ependymal E1 and E2 cells. Astrocyte, microglial, and intermediate progenitor populations were also studied in dorsal SVZ. DAPI labelling confirms an increase in cell density analyzed relative to the length (μm) and total area (μm^2^) of the dorsal SVZ (Fig. 2a, d). This increase was associated with GFAP occupying a larger area of this tissue (Fig. 2b, e) and by an increase in the total number of Iba1^+^ cells/mm^2^ (Fig. 2c, f). Some minor changes to the morphology of the microglia in the dorsal SVZ were evident, whereas no such differences were evident in the corpus callosum (Fig. 2g).

We next examined the intermediate DCX^+^ progenitor cells in the dorsal SVZ and no changes in the area occupied by these cells were produced by TMEV infection (Fig. 2h, i). Imaris reconstructions revealed a heightened intensity of DCX labelling in the corpus callosum compared to the SVZ, probably related to an increase of this microtubule-associated protein in migrating progenitors that exit the SVZ toward the demyelinated areas (see the isolated DCX^+^ cell in the corpus callosum with a typical migratory morphology, consisting in a leading process orientated toward the direction of migration: Fig. 2j). An analysis of the area occupied by DCX^+^ cells and the intensity of their labelling in the demyelinated corpus callosum revealed a relative decrease in both parameters in TMEV mice (Fig. 2k, i).

**Fig. 3:**
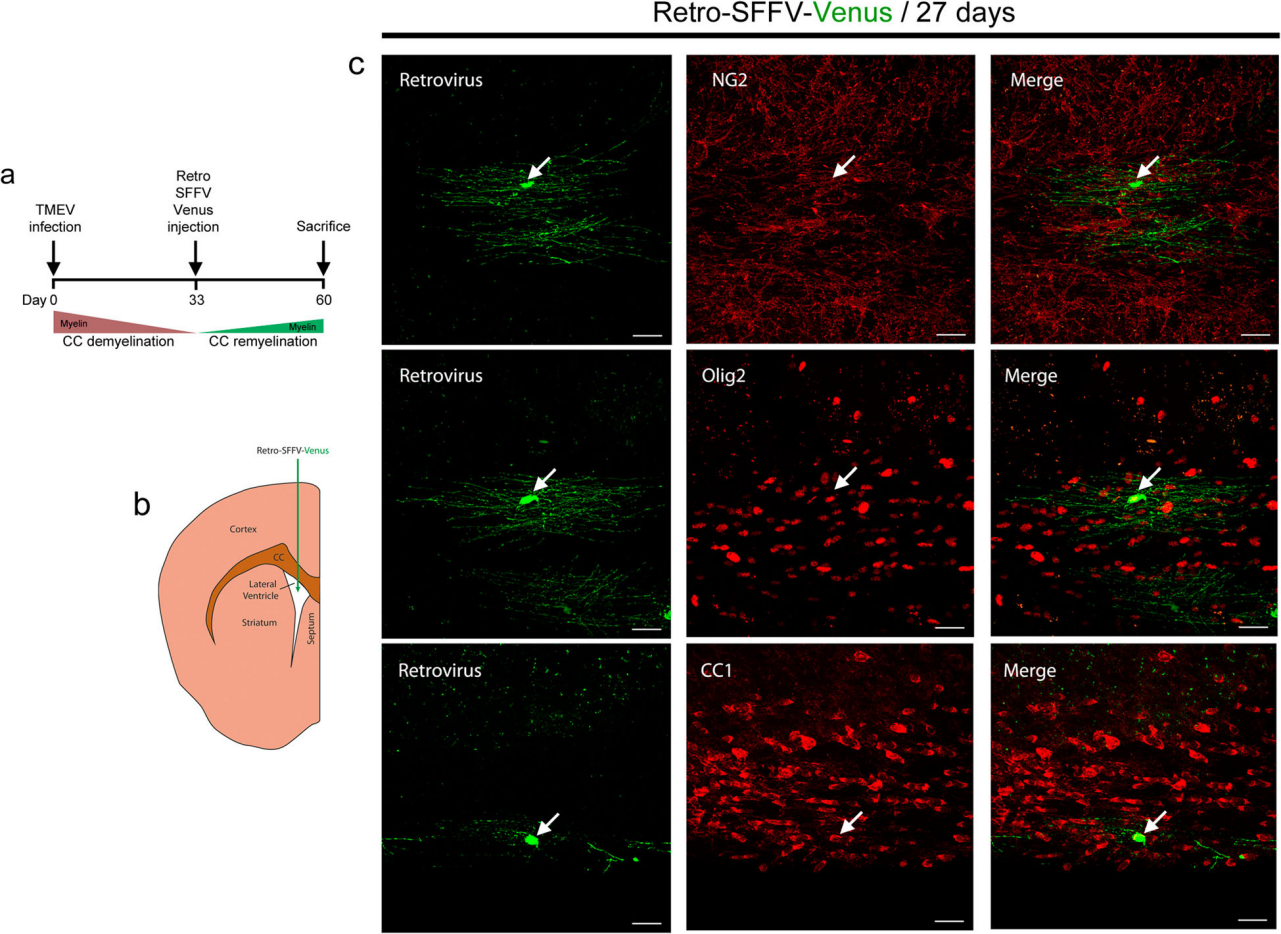
Proliferating SVZ progenitors produce oligodendrocytes that contribute to corpus callosum remyelination in the TMEV-IDD model. **a**, **b** Scheme of the intraventricular administration of the SFFV-Venus retroviral vectors at the onset of corpus callosum remyelination to track the labeled, proliferating progenitors in the SVZ. **c** Green Venus^+^ cells can be found in the remyelinated corpus callosum at 27 days p.i. The oligodendroglial identity of these cells, with a typical branched myelinating morphology, was evaluated by immunostaining, showing that they are negative for the OPC marker NG2, and positive for the mature oligodendrocyte markers Olig2 and CC1. Scale bar: 25 μm. Images are representative of three independent experiments

To further evaluate the contribution of the SVZ to corpus callosum remyelination, we took advantage of the fluorescent retroviral vector (LentiGO), which only labels cells that are actively proliferating, including SVZ progenitors. These vectors have been shown to track glial progenitors originated from the SVZ [30]. SFFV-Venus γ-retroviral particles were injected into the lateral cerebral ventricle on day 33 p.i. (Fig. 3a, b) and the progeny of the SVZ proliferative progenitors mobilized was studied in the corpus callosum 27 days later, highlighting the presence of Venus^+^ oligodendrocytes that did not express the OPC marker NG2 and but that did express the mature oligodendrocyte markers CC1 and Olig2 (Fig. 3c).

### Wnt proteins are differentially expressed during corpus callosum remyelination

**Fig. 4:**
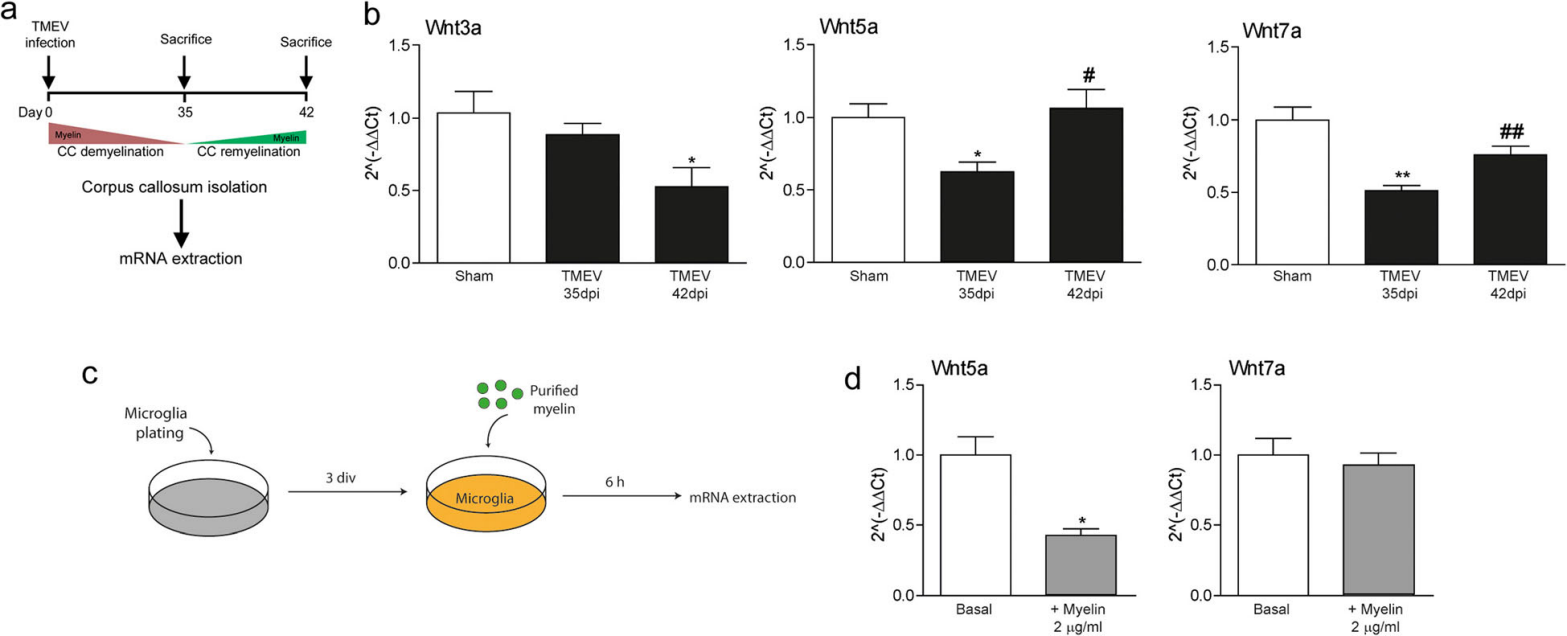
Wnt gene expression are differentially regulated during corpus callosum remyelination and in microglia exposed to myelin. **a**, **b** Tissue dissection to evaluate Wnt3a, Wnt5a and Wnt7a gene expression in the demyelinated corpus callosum (day 35 p.i.), and during remyelination (day 42 p.i.) *N* = 6–10 mice per experimental group. Statistics: *p ≤ 0.05; **p ≤ 0.01 vs. Sham; #*p* ≤ 0.05; ##*p* ≤ 0.01 vs. TMEV day 35 p.i. (post-infection); ANOVA followed by Bonferroni’s post-hoc test. **c**, **d** In vitro analysis of Wnt5a and Wnt7a gene expression of microglia following the addition of purified myelin. *N* = 6 independent experiments with three replicates. Statistics: *p ≤ 0.05 vs. basal; Student’s *t* test

Active MS lesions express multiple elements of the Wnt signaling pathways not evident in chronic silent plaques or in the apparently normal white matter [39]. During remyelination, Wnt glycoproteins can affect the differentiation and maturation of OPCs and other precursors (e.g., progenitor cells) that migrate from the SVZ to generate new oligodendrocytes and myelin sheaths. We first analyzed the expression of genes encoding canonical and non-canonical Wnt ligands in the demyelinated corpus callosum, and during active remyelination (day 35 and 42 p.i., Fig. 4a). In the demyelinated corpus callosum of TMEV-infected mice, Wnt3a expression on day 35 p.i. was not modified relative to the Sham mice in the RT-PCR analysis, whereas less Wnt5a and Wnt7a mRNA was expressed (Fig. 4b). During active remyelination, Wnt3a downregulation was concomitant to an upregulation of Wnt5a and Wnt7a gene expression, suggesting different roles for Wnt signaling during remyelination.

### Myelin alters the expression of the Wnt5a gene by microglia, and it stimulates the generation and maturation of oligodendrocytes from NSCs

Microglia are pivotal cells in corpus callosum remyelination, controlling the phagocytosis and clearance of myelin debris [25]. As Wnt glycoproteins can influence microglia functions [40, 41], we wanted to assess whether myelin affects Wnt gene expression by microglia in vitro. Microglial cells in culture were exposed to purified myelin (2 μg/ml) and the expression of Wnt3a, Wnt5a, and Wnt7a was analyzed by RT-PCR (Fig. 4c). While no Wnt3a expression was detected (data not shown), Wnt5a was expressed less strongly by microglia in the presence of myelin in the absence of any effect on Wnt7a gene expression (Fig. 4d).

**Fig. 5:**
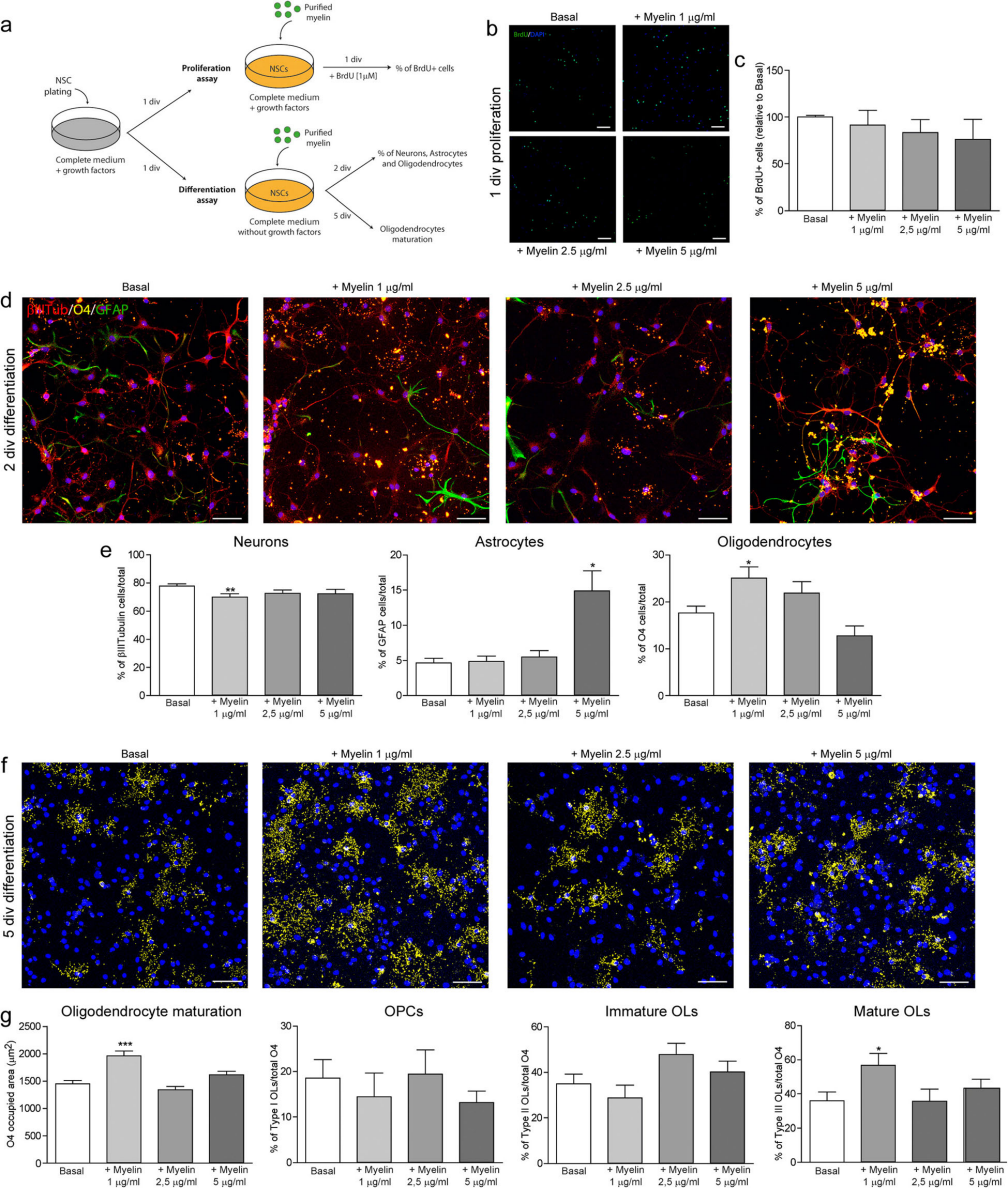
Myelin contributes to the generation and maturation of oligodendrocytes from NSCs. **a** Scheme of the in vitro assays performed to evaluate the effects of purified myelin on the proliferation and differentiation of NSCs. **b**, **c** BrdU labelling and the proliferation of NSCs following the addition of purified myelin (1, 2.5, or 5 μm). **d** Triple immunocytochemistry performed after 2 days of NSCs differentiation to identify neurons (β-III Tubulin, red), astrocytes (GFAP, green), and oligodendrocytes (O4, yellow), as analyzed in (**e**). **f** O4 labelling performed after five div in the presence of purified myelin (1, 2.5, or 5 μm), and analyzed in (**g**) as the total area occupied by O4, and the proportion of OPCs, immature and mature oligodendrocytes. *N* = 3 independent experiments. Statistics: *p ≤ 0.05; **p ≤ 0.01; ***p ≤ 0.001 vs. Basal; ANOVA followed by Bonferroni’s post-hoc test. Scale bar: 50 μm

Contact with central nervous system (CNS) myelin appears to inhibit the maturation of immature A2B5^+^ OPCs in vitro [42]. Since CNS myelin could also influence the differentiation and maturation of intermediate progenitors that migrate from the SVZ and encounter an inhibitory environment in the demyelinated corpus callosum, we studied whether myelin affected the generation and maturation of oligodendrocytes derived from NSCs in vitro. NSCs in culture were exposed to purified myelin (1, 2.5, and 5 μg/ml, see Fig. 5a), and the proliferation (24 h) and differentiation (2 and 5 days in vitro -div) of NSCs and generated oligodendrocytes was analyzed. CNS myelin did not affect the proliferation of NSCs as the proportion of cells that incorporated BrdU^+^ was similar to that seen in basal conditions (Fig. 5b, c). NSCs spontaneously developed into neurons (β-III Tubulin^+^), astrocytes (GFAP^+^), and oligodendrocytes (O4^+^) after two div (Fig. 5d, e), yet the lowest dose of myelin (1 μg/ml) diminished the proportion of neurons, concomitant with an increase in the percentage of oligodendrocytes derived from NSCs. By contrast, in the presence of the highest dose of CNS myelin (5 μg/ml), an increase was only observed in the proportion of astrocytes generated. After five div in the presence of the lowest dose of CNS myelin (1 μg/ml), the area occupied by O4^+^-labeled cells and the proportion of mature oligodendrocytes was enhanced, parameters that were not affected in the presence of the higher doses of myelin (Fig. 5f, g).

### Wnt7a induces the generation of oligodendrocytes from NSCs

**Fig. 6:**
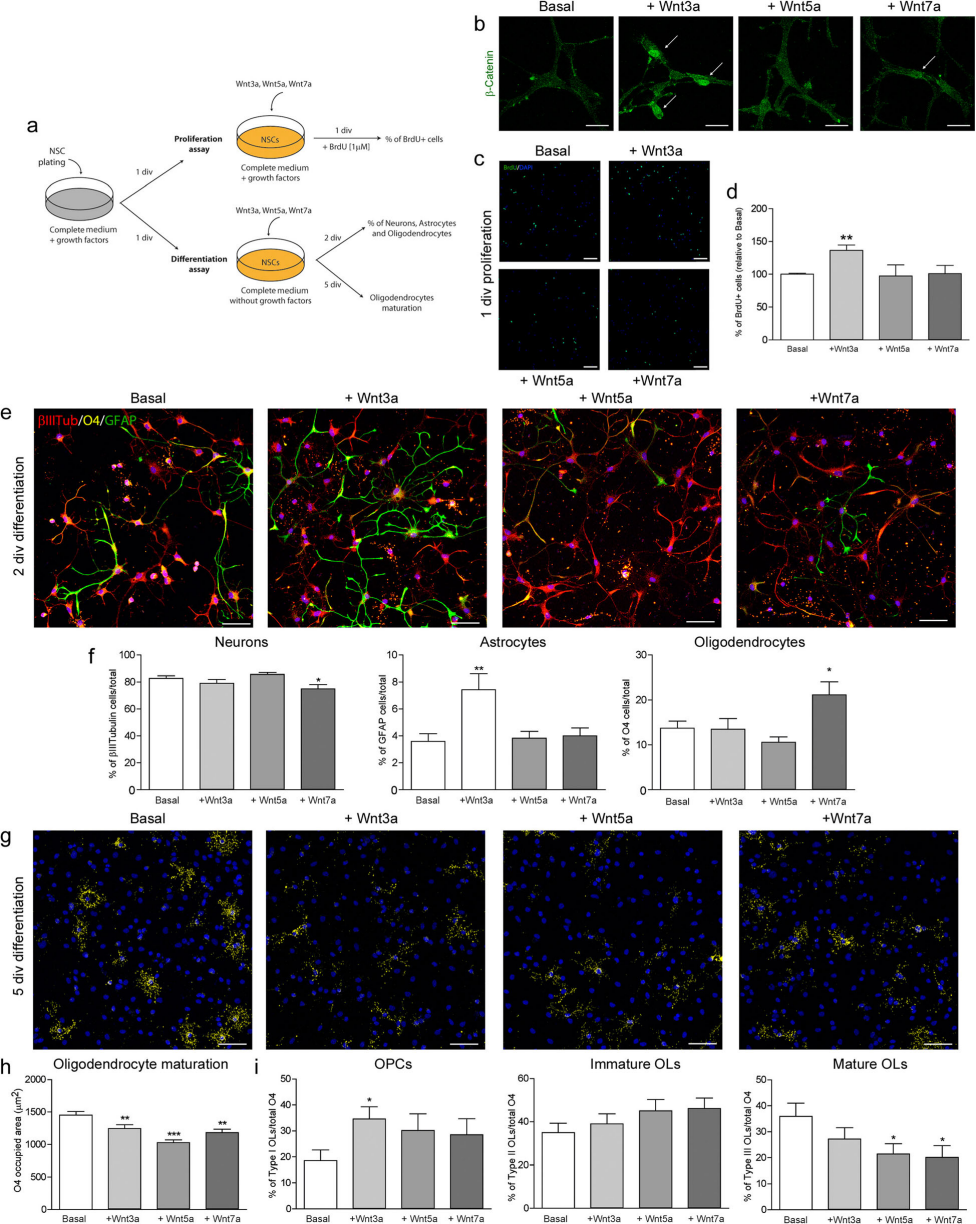
Effects of Wnt3a, Wnt5a and Wnt7a on the generation of oligodendrocytes from NSCs. (**a**) Scheme of the in vitro assays performed to evaluate the effects of Wnt3a, Wnt5a and Wnt7a on the proliferation and differentiation of NSCs. (**b**) b-Catenin labelling following the addition of Wnt proteins. (**c**, **d**) BrdU labelling and the proportion of proliferative NSCs following the addition of Wnt proteins (200 ng/mL). (**e**) Triple immunocytochemistry performed after 2 days of NSCs differentiation to identify neurons (b-III Tubulin, red), astrocytes (GFAP, green) and oligodendrocytes (O4, yellow), and analyzed in (**f**). (**g**) O4 labelling performed after 5 div in the presence of Wnt proteins (200 ng/mL), and analyzed in (**h**) as the total area occupied by O4 and, the proportion of OPCs, Immature and Mature oligodendrocytes. *N* = 3 independent experiments. Statistics *p ≤ 0.05; **p ≤ 0.01; ***p ≤ 0.001 vs Basal; ANOVA followed by Bonferroni´s post-hoc test. Scale bar: 25 mm in **a**; 50 mm in **c**, **e**, **g**

The proliferation and differentiation of NSCs on exposure to Wnt3a, Wnt5a, and Wnt7a (200 ng/ml) was assessed in vitro (see scheme in Fig. 6a). The activation of canonical (Wnt3a, Wnt7a) and non-canonical (Wnt5a) pathways by these ligands was confirmed by β-Catenin labelling, an increase in nuclear β-Catenin staining evident 24 h after stimulation with Wnt3a and Wnt7a (Fig. 6b). BrdU incorporation revealed a proliferative effect of Wnt3a on NSCs (Fig. 6d), which resulted in an increase in GFAP^+^ astrocytes after two div (Fig. 6e, f). Wnt5a did not induce any effect on the proliferation or differentiation of NSCs, whereas Wnt7a significantly reduced the differentiation of NSCs into neurons while increasing the number of oligodendrocytes generated (Fig. 6e, f). After five div, the differentiation and maturation of NSCs stimulated with Wnt3a, Wnt5a, or Wnt7a into O4^+^ oligodendrocytes was delayed when compared to the basal conditions (Fig. 6g, h). Wnt3a increased the proportion of OPCs, while both Wnt5a and Wnt7a decreased the percentage of mature oligodendrocytes (Fig. 6i).

### M1 microglia secrete the Wnt5a protein, while M2c microglia secrete Wnt7a

**Fig. 7:**
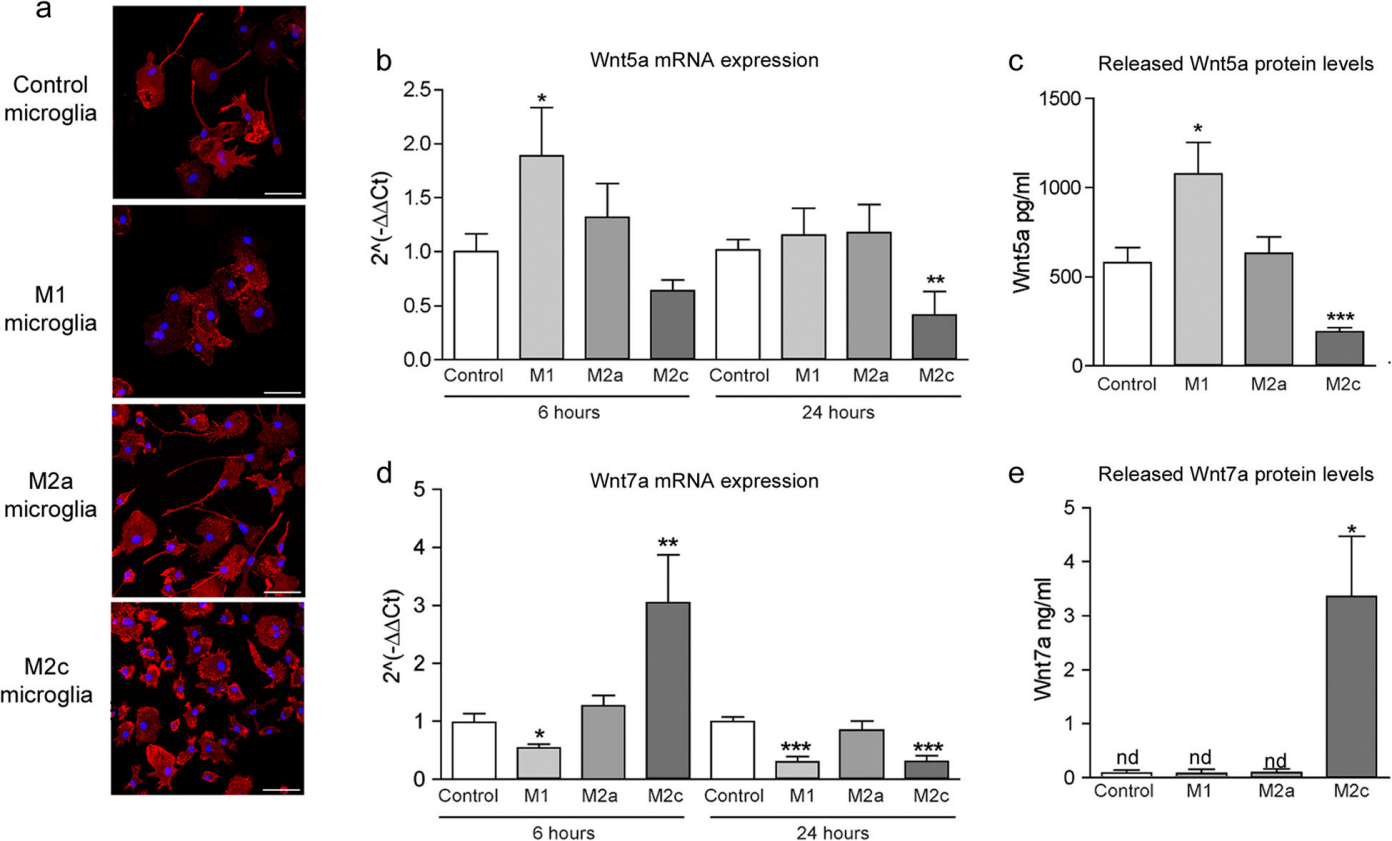
M1 microglia secrete Wnt5a, while M2c microglia secrete the Wnt7a protein. **a** OX-42 staining of microglial cells following 24 h of stimulation with polarization agents to obtain the M1, M2a, and M2c activation states. **b**, **d** Wnt5a and Wnt7a gene expression by microglia in different activation states after 6 and 24 h of polarization. **c**, **e** Wnt5a and Wnt7a protein secreted after 24 h of polarization, evaluated in the supernatant of the cultures by ELISA. *N* = 4 independent experiments with three replicates. Statistics: *p ≤ 0.05; **p ≤ 0.01; ***p ≤ 0.001 vs. Control; ANOVA followed by Bonferroni’s post-hoc test. Scale bar: 25 μm

While several soluble factors released by microglia can direct neural precursor cell migration and differentiation, including cytokines and growth factors [21], the secretion of Wnt proteins by microglia has yet to be assessed. We evaluated this by polarizing microglia into the M1, M2a, and M2c activation states in vitro (Fig. 7a), and then, analyzing the expression of Wnt5a and Wnt7a mRNA and protein (note that Wnt3a was not detected in polarized microglia in RT-PCR studies, data not shown). Compared to unstimulated cells, M1 microglia transiently upregulated their Wnt5a expression, which in turn provoked an increase in the release of this glycoprotein into the culture medium (Fig. 7b, c). The opposite effect was found in M2c microglia, in which the decrease in Wnt5a mRNA 24 h after stimulation was associated with a decrease in the Wnt5a protein released. No changes were found in M2a polarized microglia in relation to the Wnt5a mRNA or protein levels. By contrast, M2c microglia upregulated the expression of Wnt7a and its secretion (Fig. 7d, e), whereas M1 microglia dampened their Wnt7a expression. Similarly, M2a microglia showed no changes in the mRNA expression or protein levels of this glycoprotein.

### M2c microglia conditioned medium induces the generation of oligodendrocytes from NSCs

**Fig. 8:**
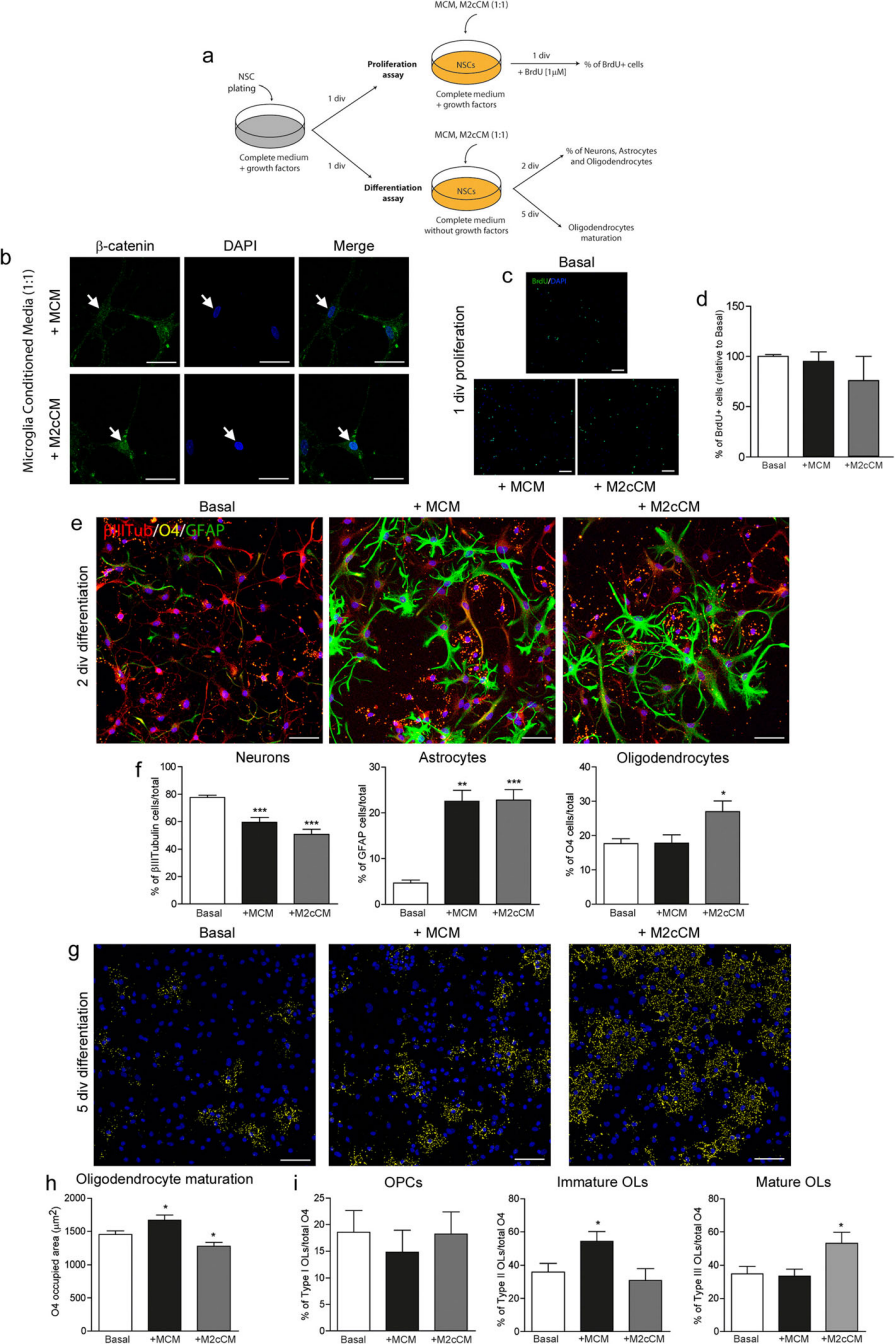
M2c microglia conditioned medium contributes to the generation of oligodendrocytes from NSCs. (**a**) Scheme of the in vitro assays performed to evaluate the effects of conditioned media from control (MCM) and M2c (M2cCM) microglia on the proliferation and differentiation of NSCs. (**b**) b-Catenin and DAPI labelling following the addition of MCM or M2cCM to NSCs. (**c**, **d**) BrdU labelling and proportion of proliferative NSCs 1 div after the addition of MCM and M2cCM (1:1). (**e**) Triple immunocytochemistry performed after 2 days of NSC differentiation to identify neurons (b-III Tubulin, red), astrocytes (GFAP, green) and oligodendrocytes (O4, yellow), and analyzed in (**f**). (**g**) O4 labelling performed after 5 days of culture in the presence of MCM and M2cCM (1:1), and analyzed in (**h**) as the total area occupied by O4 and the proportion of OPCs, Immature and Mature oligodendrocytes. *N* = 3 independent experiments. Statistics: *p ≤ 0.05; **p ≤ 0.01; ***p ≤ 0.001 vs Basal; ANOVA followed by Bonferroni´s post-hoc test. Scale bar: 25 mm in **a**; 50 mm in **c**, **e**, **g**

Given the oligodendrogenic effects of the Wnt7a produced and secreted by M2c microglia on NSCs, we evaluated the effect of medium conditioned by these microglia on the proliferation and differentiation of NSCs (see scheme in Fig. 8a). The nuclear β-catenin staining confirmed that the canonical Wnt/β-catenin pathway was activated in NSCs cultured in conditioned medium from M2c microglia (M2cCM) relative to the unstimulated control microglia (MCM, Fig. 8b). MCM or M2cCM had no effect on the proliferation of NSCs (Fig. 8c, d), yet these media reduced the proportion of neurons while they enhanced the presence of astrocytes relative to the basal NSC medium. Furthermore, M2cCM promoted the production of oligodendrocytes (Fig. 8e, f). Finally, MCM significantly increased the area occupied by O4^+^ cells and the percentage of immature oligodendrocytes, while M2cCM decreased the area occupied by O4^+^ cells and it increased the proportion of mature oligodendrocytes (Fig. 8h, i).

## Discussion

Myelin remodeling in the adult CNS requires the differentiation of oligodendrocytes in order to generate myelin sheaths around naked axons. New mature oligodendrocytes mainly originate from local precursors distributed along the nervous parenchyma, although the involvement of the SVZ niche must be considered when contemplating remyelination in adjacent white matter areas like the corpus callosum. To study the contribution of the dorsal SVZ niche to corpus callosum remyelination, we used the viral TMEV-IDD model that resembles MS pathogenesis, involving inflammation, demyelination, and neurodegeneration [43, 44]. In this model, there is prominent corpus callosum demyelination 35 days p.i. in parallel with SVZ proliferation [38] due to the prominent inflammatory reaction against the virus known as acute encephalomyelitis [26]. In addition, we used in vitro approaches to analyze the effect of local signals present in the demyelinated corpus callosum on NSC proliferation and differentiation, including myelin debris, Wnt signaling, and activated microglia.

In the adult SVZ, the presence of B1 NSCs contacting the ventricles confers an unusual cytoarchitecture to the ventricle wall, where the apical surfaces of B1 cells are surrounded by ependymal E1 and E2 cells in a pattern similar to a pinwheel that is fundamental for adult neurogenesis [31]. Using whole mount preparations to study this cytoarchitecture, we analyzed the number of NSCs and the pinwheel distribution at the dorsal wall of the lateral ventricle in TMEV-infected mice at day 35 p.i. As in our previous work [38], the B1 cells in the dorsal SVZ increased significantly, together with an increase in GFAP staining and cell density. The number of pinwheels also augments, supporting the activation of the dorsal SVZ in response to corpus callosum demyelination. This is consistent with other reports showing an increase in B1 cell proliferation after inflammatory demyelination [45, 46], and the activation of the SVZ following cuprizone demyelination [47], TMEV infection [48], and in MS patients [7]. The contribution of SVZ precursors to corpus callosum remyelination has been addressed in the EAE model of MS [9] and after toxic demyelination, using in vivo genetic fate mapping [13], single-cell tracking with β-actin GFP [11] and retroviral GFP injections [49]. Here, in vivo γ-retroviral vectors facilitated the single-cell tracking of newborn adult oligodendrocytes originating from B1 proliferative cells in the dorsal SVZ, indicating the contribution of this niche to corpus callosum remyelination in the TMEV-IDD model. Concomitant to dorsal SVZ activation, the intensity and area occupied by DCX precursors in the demyelinated corpus callosum diminished but not in the dorsal SVZ. The glial fate of DCX^+^ progenitors that contribute to the generation of new oligodendrocytes in the corpus callosum has been addressed after lysolecithin injection [50], with a loss of this marker as precursors start to express mature oligodendrocyte markers [51]. Hence, together with the mobilization and activation of local OPCs following demyelination [25], some signals from the damaged corpus callosum cause stem cells in the dorsal SVZ to proliferate, inducing the generation of DCX^+^ oligodendrocyte intermediate progenitor cells (oIPCs) that are not committed to the neuronal lineage but rather, that migrate to the lesion site and differentiate into mature oligodendrocytes.

Demyelination is associated with the generation of myelin debris that impairs OPC differentiation [27, 42], although myelin direct effect on NSC differentiation has not yet been studied. Here, we show that purified myelin may promote the generation of oligodendrocytes from NSCs, supporting their differentiation in vitro. We speculate that the myelin debris generated in the demyelinated corpus callosum not only affects local OPCs but also the SVZ niche, contributing to the specification of oIPCs committed to the oligodendroglial fate and their differentiation into mature oligodendrocytes. In this scenario, we found a dysregulation of Wnt expression following demyelination in TMEV-IDD mice, with a decrease in Wnt5a and Wnt7a mRNA transcripts in the demyelinated corpus callosum and a decrease in Wnt3a during remyelination. The temporal discordance in Wnt expression would suggest a role for these proteins in NSC proliferation in the dorsal SVZ and the differentiation of these cells in the demyelinated corpus callosum. The dorsal SVZ is enriched in pro-oligodendrogenic signaling molecules like those in the Wnt/β-catenin pathway, indicating a greater capacity to generate oligodendrocytes than the lateral or medial SVZ [15]. Wnt signaling has a canonical and a non-canonical branch depending on the downstream involvement of the β-catenin pathway [52]. A shift from the non-canonical to canonical Wnt pathway has been associated with the activation of quiescent NSCs in the lysolecithin model of focal demyelination [53], and β-Catenin signaling is active during remyelination [54]. Indeed, canonical β-catenin dependent [55] and non-canonical β-catenin independent [53] Wnt signaling has been attributed a crucial role in remyelination, although this issue remains controversial [55]. Furthermore, active MS plaques express Wnt signaling genes more strongly and accumulate more Wnt protein than chronic lesions and normal appearing white matter [39, 56]. Here, canonical Wnt3a promotes NSC proliferation in vitro, whereas canonical Wnt7a enhances the production of oligodendroglial cells from NSCs. Although Wnt5a may activate β-catenin signaling, predominantly activates non-canonical cascades as planar cell polarity (Wnt5a/JNK route) and calcium pathway (Wnt5a/Ca2^+^) [57]. Indeed, Wnt5a produced no effects on NSCs in our cultures. Moreover, Wnt3a can enhance sub-ependymal zone cell proliferation in culture, in conjunction with the specification of these cells toward NG2^+^ OPCs [58]. Actually, direct effects of Wnt proteins on OPCs should be further considered during remyelination, since these proteins play multiple roles in CNS development, adult oligodendrogenesis [59], and during myelinogenesis [60].

Microglial activation mediates activities that range from protection against harmful conditions to the restoration of CNS homeostasis following inflammation. Studies have begun to realize that alterations in microglia immune phenotypes, gene protein expression, and morphology are highly complex processes that exist on a broad spectrum [61, 62]. Actually, microglia activation is not a completely polarized M1 or M2 activation state with clear differentiated expression of inflammatory mediators but a balanced one, with higher levels of pro- or anti-inflammatory molecules depending on the stimuli which define the actions and function of microglia [61]. Despite the limitations of applying the M1/M2 framework to microglia, in neuroinflammatory situations such as in TMEV-IDD and focusing on microglia, we found different activation dynamics along time that include and initial pro-inflammatory shift followed by an anti-inflammatory shift. Activated microglia secreting pro-inflammatory cytokines, chemokines, and nitric oxide can be found in the initial acute antiviral responses (7–21 days p.i.) as well as in the beginning of the chronic phase of the disease (70–85 d.p.i.) [26]. Between both M1 activation profiles, microglia acquired an alternatively activated state M2 characterized by the limited secretion of pro-inflammatory cytokines, the production of factors involved in repair and tissue reconstruction, and that of anti-inflammatory cytokines [63]. M2c microglia also mediate the clearance of myelin debris, as well as synthesizing pro- and anti-inflammatory cytokines in the corpus callosum following demyelination in TMEV-IDD mice, resulting in partial remyelination [25]. Here, we show that concomitant to dorsal SVZ activation, there is an increase in the number of microglial cells with some minor morphological changes in this area. Microglial cells residing in the SVZ exhibit characteristics of an alternatively activated state, supporting adult neurogenesis, proliferation, and migration [18]. Indeed, pro-oligodendrogenic effects of microglia have been described in the early postnatal SVZ [64] and in the adult SVZ following nearby demyelination [47, 65]. Selective depletion of microglia suggests that the direct contact of stem cells with microglia is not necessary for neurogenesis. Soluble factors present in microglia-conditioned media from the SVZ or cerebellum can drive neurogenesis rather than supporting NSC maintenance [20]. Thus, microglia may instruct stem cells to contribute to remyelination by secreting soluble signals that promote the oligodendroglial fate. Moreover, we found that the addition of purified myelin to microglia cultures diminished the expression of Wnt5a but not of Wnt7a. Since microglia express Wnt receptors and Wnt3a per se is pro-inflammatory [40], whereas Wn3a/Wnt5a counteracts the pro-inflammatory actions of LPS in these cells [41], the activation state of microglia could be related not only to the differential effects of Wnts but also to the specific production and secretion of Wnt proteins by these cells.

The secretion of pro-inflammatory cytokines by microglia, including IL-1β, IL-6, TNF-α, and IFN-γ, promotes neurogenesis and oligodendrogenesis in the SVZ, and in co-cultures in vitro [64]. Depending on the activation state of microglia, their secretome produces different effects on the survival, migration, and differentiation of NSCs [66]. Specifically, conditioned medium from M2a microglia promotes neurogenesis and oligodendrogenesis of NSCs through PPARγ signaling [67]. Here, we describe the selective synthesis and secretion of Wnt proteins depending on the activation state of microglia, with M1 microglia secreting Wnt5a and M2c microglia secreting Wnt7a, while no detectable levels of Wnt3a mRNA were found irrespective of the microglial activation state. We focused on M2c microglia to further analyze the effects of this activation state on NSCs, conditioned media from these cells enhancing the β-Catenin in the nucleus of NSCs, suggesting the activation of the canonical Wnt7a/β-Catenin pathway. Furthermore, M2c conditioned medium promoted oligodendrocyte differentiation from NSCs when compared to the non-polarized microglia or basal conditions, reducing the area occupied by O4^+^ cells, in accordance with the direct effects when NSCs are exposed to Wnt7a in culture. This finding highlights the importance of Wnt7a in the secretome of M2c microglia, suggesting new functions of these cells in neurogenesis depending on their activation state. Although we did not find any effect of Wnt5a on the proliferation or specification of NSCs, the effects of pro-inflammatory microglia via the non-canonical Wnt pathway in NSCs should still be further explored.

## Conclusions

**Fig. 9:**
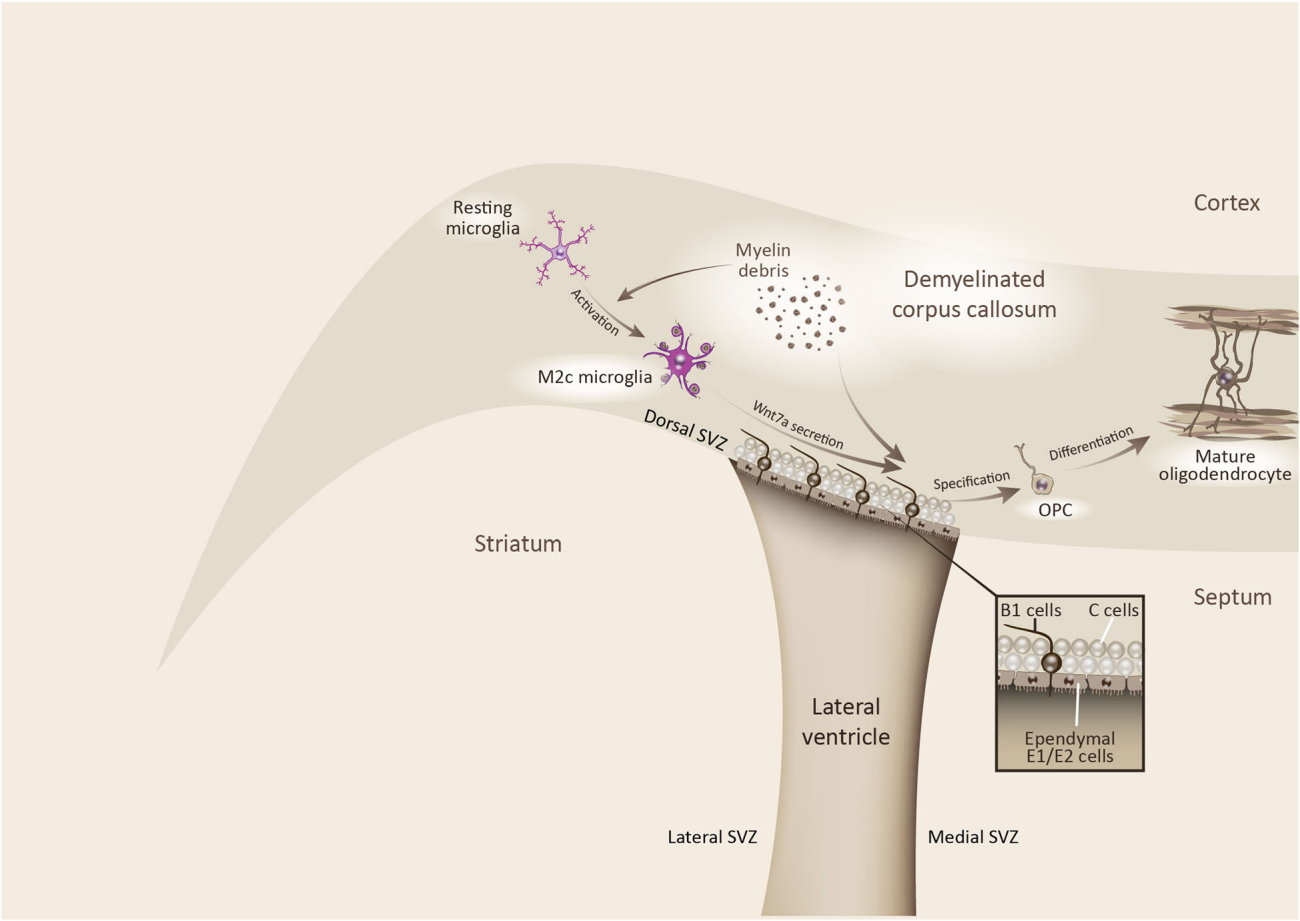
Myelin debris and M2c microglia contribute to oligodendrocytes generation from dorsal SVZ stem cells. Coronal mouse brain reflecting the capacity of the corpus callosum and dorsal SVZ to generate oligodendrocytes via environmental signals. The myelin debris generated during demyelination provokes microglial activation towards the phagocytic M2c state that secretes the Wnt7a protein. The Wnt7a/β-Catenin pathway drives the specification of dorsal SVZ stem cells towards an oligodendrocyte intermediate progenitor cell (oIPC) fate, committed to the oligodendroglial lineage. Conversely, myelin debris directly affects the specification and differentiation of oIPCs towards mature oligodendrocytes. Oligodendrogenesis may not only be mediated by the mobilization and differentiation of local OPCs but also, by the specification and differentiation of stem cells in the dorsal SVZ through the activity of M2c microglia and the collateral effects of the myelin debris generated during demyelination

In conclusion, our data suggest that the myelin debris generated during corpus callosum demyelination can directly affect the specification and differentiation of multipotential cells in the dorsal SVZ (see Fig. 9). Stem cells generate oIPCs that are committed to the oligodendroglial lineage and that migrate to the lesion site, where they differentiate into mature remyelinating oligodendrocytes. Myelin breakdown also induces the activation of microglia from a resting state toward an M2c phagocytic state. M2c cells secrete a plethora of factors, including Wnt7a that can influence the specification of dorsal SVZ multipotential cells to the oligodendroglial lineage. Hence, these data reinforces the role of M2c microglia in NSC oligodendrogenesis which may be driven through the canonical Wnt7a signaling pathway among others factors. Our study also evidences the possible role of microglia in adult oligodendrogenesis upon demyelination, targeting local OPC populations and NSC progenitors in the SVZ.

## Data Availability

The raw data supporting the conclusions of this manuscript will be available by the authors, without undue reservation, to any qualified researcher.

## Abbreviations

CNS: Central nervous system
DCX: Doublecortin
M2cCM: M2c conditioned media
MCM: Microglia-conditioned media
MS: Multiple sclerosis
NSCs: Neural stem cells
oIPC: Oligodendrocyte intermediate progenitor cell
OPC: Oligodendrocyte progenitor cell
RMS: Rostral migratory stream
SVZ: Subventricular zone
TMEV-IDD: Theiler’s murine encephalomyelitis virus-induced demyelinating disease
